# Direct and Indirect Cationization of Cellulose Nanocrystals: Structure–Properties Relationship and Virus Capture Activity

**DOI:** 10.1021/acs.biomac.2c01045

**Published:** 2022-12-05

**Authors:** Maryam Madani, Sedigheh Borandeh, Arun Kumar Teotia, Jukka V. Seppälä

**Affiliations:** Polymer Technology, School of Chemical Engineering, Aalto University, Kemistintie 1, Espoo, 02150, Finland

## Abstract

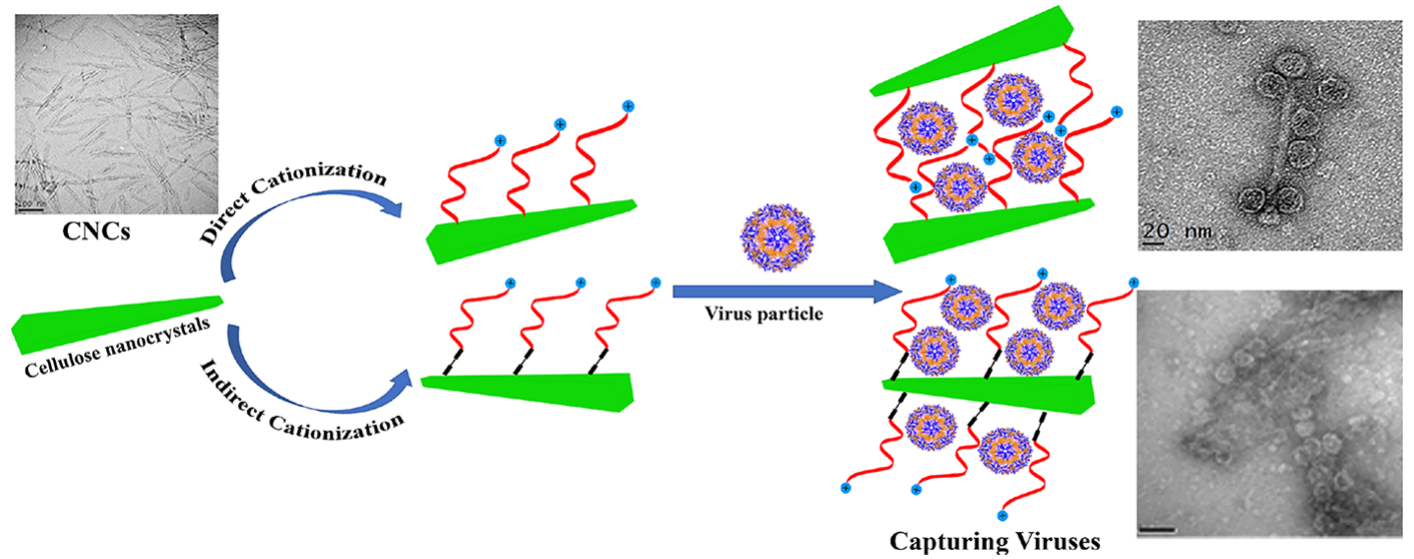

Due to increasing public concern over hygiene, there have been many studies investigating antimicrobial and antiviral agents recently. With the aim of developing biobased virucidal/virus capture agents, we report a chemical modification of the cellulose nanocrystals (CNCs) surface with poly(2-dimethylamino) ethyl acrylate) methyl chloride quaternary salt (Q-PDMAEA) to introduce the positively charged functional groups. The surface of CNCs was modified through direct and indirect graft polymerization. Subsequently, the direct and indirect cationization effect on the degree of functionalization, thermal stability, crystallinity, and antiviral activity of CNCs was investigated. Indirect cationization produced the highest degree of polymer grafting, increasing particle size and thermal stability. Further, the modified CNCs were tested for their ability to capture nonenveloped bacteriophages PhiX174 (ΦX174) and MS2. We observed a significant (>4.19 log_10_) reduction in total viral load by specific functionalized CNCs. However, the activity depended on the structure of functional groups, surface charge density, and the type of virus under study. Overall, the direct and indirect cationization of CNC leads to biobased agents with immobilized cationic charge, with good virus capture activity. Such agents can be used for various applications including textiles, packaging, wastewater treatment, etc.

## Introduction

Cellulose nanocrystals (CNCs) are rod-shaped crystals (∼100 nm long) that are formed by hydrolyzing cellulose, the most abundant polysaccharide.^1^ As a result of their excellent characteristics like high specific aspect ratio, sustainability, and biodegradability, CNCs have become increasingly popular for a variety of applications, especially in biomedicine.^2,3^

Additionally, the growing need for high-performance materials with antimicrobial properties has led to CNCs becoming the most attractive renewable material, providing template for different surface modifications.^3−7^ Most viruses and bacteria under normal physiological conditions contain a net negative charge, a material containing opposite (cationic) charge can effectively capture or neutralize such bacteria and viruses via charge–charge interactions.^8,9^ The amount of charge carried by a virus depends on the total amount of charge carried by both the genetic material and the protein.^10^

Researchers have explored the possibility of creating antimicrobial materials with cationic functional groups deposited on CNCs surface.^11−14^ The presence of a high number of hydroxyl groups on the CNCs surface makes it an ideal candidate for such functionalizations.^15,16^ Due to their abundance, sustainable origin, biocompatible, and nontoxic nature, cationic CNCs have been widely prepared.

Several recent studies prepared cationic CNCs by grafting either cationic small molecules or by cationic polymers. Hasani et al.^17^ used (2,3-epoxypropyl) trimethylammonium chloride as the cationic agent for an etherification reaction with activated hydroxyl groups of CNCs. The cationization of CNCs decreases the anionic charge of CNCs and improves its aqueous suspension stability. Studies, such as those by Lin et al.^18^ and You et al.,^19^ prepared cationic CNCs for biomedical applications. In one of them biocompatible double-membrane hydrogels were prepared using cationic CNCs. In another study injectable nanocomposite hydrogels based on quaternized cellulose fibrils and cationic CNCs were prepared for sustained delivery of anticancer drugs.^18,19^

Graft polymerization is one of the widely used approaches for modification of chemical and physical properties of CNCs.^20−22^ For instance, Liu et al.^23^ and Hemraz et al.^24^ have extensively investigated surface-initiated grafting polymerization reactions onto the surface of CNCs. Their approaches involved several steps for grafting of cationic polymers. A quaternized CNC was first prepared by coating the surface with α-bromoisobutyryl bromide initiator, then polymerizing acrylate monomer using radical polymerization, and finally quaternizing the polymer onto the CNCs surface. An easy approach of attaching polymers on CNCs surfaces would be via radical graft polymerization with vinyl monomer. Several studies have used vinyl monomers for such modification of CNCs by using thermal initiators like potassium persulfate or ammonium persulfate (APS). For example, 2-(3-(6-methyl-4-oxo-1,4-dihydropyrimi-din-2-yl)ureido)ethyl methacrylate,^25^ poly[2-(dimethylamino)ethyl methacrylate],^26^ and poly(acrylic acid)^27^ were successfully polymerized on the CNCs surface using these initiators. The main advantage of using this method is that the grafting can be performed directly from pristine CNCs in one step without any modifications beforehand.

To our knowledge, until now, most studies have focused on either cationization with small molecules or graft polymerization of CNCs and investigated their antiviral/antibacterial properties.^12,28^ However, we found no studies where the effect of direct and indirect cationic grafting of CNC surface on their antiviral activity had been carried out. In this study, we aimed to develop a biobased antiviral compound through graft polymerization of a dual functional quaternary ammonium vinyl monomer, [2-(acryloyloxy)ethyl] trimethylammonium chloride solution (AETAC) on CNCs. It can be polymerized on the surface of CNCs via an acrylic group, displaying quaternary ammonium groups that may provide antiviral properties to CNCs. The CNCs were functionalized with poly(2-dimethylamino)ethyl acrylate) methyl chloride quaternary salt (Q-PDMAEA) by graft polymerization of AETAC onto the surface of CNCs in two ways. First, AETAC was grafted onto the CNCs surface through direct free radical polymerization. In the second process, CNCs were first modified by Cystamine (Cys), followed by cationization using AETAC. Finally, the chemical, physical, and morphological characteristics of modified CNCs with Q-PDMAEA (QCNCs) were characterized using analytical, spectroscopic, and microscopic techniques. The purpose of using these two methods was to investigate the effect of using a linker between CNCs and AETAC on the degree of functionalization, thermal stability, crystallinity, and antiviral activity of QCNCs. The antiviral activity was evaluated using two different nonenveloped viruses PhiX174 (ΦX174) and MS2 and evaluate for their structure property relationships. We observed a direct correlation between the functional group density and chain flexibility on the antiviral activity of QCNCs.

## Experimental Section

### Materials

Powdered cellulose nanocrystals (CNCs) were purchased from CelluForce (density of 0.4–0.6 g.cm^–3^, viscosity of >5 mPa.s). According to the manufacturer, the CNCs extracted from woods through a sulfuric acid hydrolysis process and the crystals have dimensions nominally of 100 nm in length, 5 nm in diameter, and an aspect ratio of 20. The CNCs were determined to contain 1.4% sulfur as residual sulfate esters based on elemental analysis. [2-(Acryloyloxy) ethyl] trimethylammonium chloride solution (AETAC), ammonium persulfate (98%) (APS), cystamine dihydrochloride 96% (Cys), 1,1′-carbonyldiimidazole (CDI), triethylamine (TEA), dialysis membrane, and all other solvents were purchased from Sigma-Aldrich. Anhydrous dimethyl sulfoxide (DMSO) (99.8%) was purchased from Alfa Aesar. All compounds were used as received without any further purification.

### Direct Surface Cationization of CNCs

For direct cationization of CNCs, three different amounts of AETAC were chosen to react with CNCs hydroxyl groups to produce Q1-CNC, Q2-CNC, and Q3-CNC. The CNCs powder (1.0 g) was dispersed in 100 mL distilled water and sonicated in bath sonicator for 30 min to form a homogeneous suspension. Then, APS (0.5 g) as an initiator along with AETAC (0.9, 1.8, and 2.7 g for Q1, Q2, and Q3, respectively) were added to the suspension. The reaction was stirred at 80 °C under an N_2_ atmosphere for 3 h in an oil bath. The final product was centrifuged and washed with distilled water three times. Further purification of all QCNCs was done using dialysis bags (cellulose membrane, mw cutoff = 14,000) in flowing water for 3 days to remove any residual reactants. After that, the purified products were dried using freeze-dryer.

### Indirect Surface Cationization of CNCs

The indirect approach involved two steps. First, the hydroxyl groups of CNCs were activated with CDI to react with Cys and produce CNC-Cys.^7^ Briefly, the CNCs (1.0 g) were dispersed in anhydrous DMSO for 1 h. The suspension was purged with nitrogen for 15 min. Next, CDI (0.5 g in 3 mL of DMSO) was quickly added into the reaction mixture and stirred in an oil bath at 40 °C for 24 h. The products, after being cooled to room temperature, were diluted, centrifuged, and redispersed in DMSO. Subsequently, Cys (1.0 g in 2 mL of DMSO) and triethylamine (TEA, 0.5 mL) were added to the CNC-CDI suspension and reacted at 40 °C for 24 h. Then, the reaction mixture was diluted, centrifuged, and redispersed in distilled water, followed by several washing cycles, dialyzed against water for 1 week, and freeze-dried. The second step was further modification of CNC-Cys with AETAC (2.26 g) for Q4-CNC, following the same procedure as for direct QCNCs functionalization mentioned previously.

### Characterization

#### Fourier Transform Infrared Spectroscopy (FT-IR)

The chemical structure of CNCs, CNCs-Cys, and QCNCs were investigated at room temperature by FT-IR spectrometer (Spectrum two, PerkinElmer, UK) with an attenuated total reflectance (ATR–IR). All spectra were collected in transmission mode, with a spectrum resolution of 4 cm^–1^, 16 scans, in the range of 4000 to 500 cm^–1^.

#### Raman Spectroscopy

Raman spectroscopy measurements were carried out using Renishaw 1000 UV Raman instrument at a wavelength of 257 nm from 100 to 2500 cm^–1^ using an Ar laser source.

#### Solid-State ^13^C NMR

The chemical structure of CNC, Q2-CNC, and Q4-CNC was characterized by solid-state NMR spectrometer (Avance III 400, Bruker, Germany). The spectra were performed at room temperature, at resonance frequency of 100.61 MHz.

#### Elemental Analysis

Elemental analysis was performed by Thermo Flash Smart (Thermo Scientific, USA). All samples were frozen and lyophilized into powder and subjected to elemental analysis to quantify the degree of functionalization of CNCs.

#### X-ray Diffraction Spectroscopy (XRD)

An XRD analysis was conducted on CNCs before and after modification to examine their crystalline structure. The XRD test was performed on a Panalytical X Pert Powder XRD (Malvern, UK) with Cu–Kα radiation (λ = 1.54 Å) at 45 kV and 40 mA. The data was collected in the 2θ range from 0° to 60° with a scanning speed of 0.05° min^–1^.

#### Scanning Electron Microscopy (SEM)

The morphology of the samples was evaluated by using a scanning electron microscope Zeiss Sigma VP (Zeiss, Germany) at a voltage of 5 kV. SEM images were taken after samples were coated with 4 nm of gold palladium with a sputter coater (Leica, Germany).

#### Transmission Electron Microscopy (TEM)

The morphologies of CNCs, Q2-CNC, and Q4-CNC were observed by transmission electrical microscopy (FEI Tecnai 12, USA) at an accelerating voltage of 120 kV. The dimensions of CNCs and modified CNCs were measured using the Gatan Microscopy Suite (Gatan Inc., USA). For studying interaction between QCNCs and virus particles, the CNCs were first dispersed in phosphate buffer (20 mM, pH 6.0) and sonicated using probe sonicator. 5.0 μL of dispersed CNC was added to 4 μL of milli-Q purified water to this 1.0 μL of purified phage diluted in SM buffer (sodium chloride (100 mM), Tris (50 mM), and MgSO_4_ (8 mM) were added and mixed well by pipetting and incubated for 10 min before loading on TEM grids. Grids were negatively stained with uranyl acetate (1% w/v) and imaged at conditions used for imaging CNCs above.

#### Atomic Force Microscopy (AFM)

MultiMode AFM (Bruker, USA) with NanoScope V controller and J scanner. The scanning mode was tapping mode in air with NCHV-A probes (Bruker, Germany). Software for analysis was NanoScope 8.15 and NanoScope Analysis 1.5. Image flattening and line corrections were applied.

#### Thermogravimetric Analysis (TGA)

Thermal stability and changes in degradation associated with the modification step were assessed with TGA Q500 (TA Instruments, USA). The samples were heated from 30 to 800 °C at a rate of 10 °C.min^–1^ in a flowing nitrogen atmosphere.

#### Dispersion of QCNCs

Freeze-dried samples of CNC and QCNCs (1 wt %) were redispersed in water, DMSO, dimethylformamide (DMF), chloroform, toluene, ethanol, and water/ethanol and sonicated for 5 min on an ice bath.

#### Dynamic Light Scattering (DLS)

The hydrodynamic size and ζ-potential of virus particles and CNCs before and after modification at different pH values were measured using Zetasizer Nano ZS90 (Malvern, UK). These measurements were conducted at 25 °C using 0.01 wt % dispersion of samples in deionized water and phosphate or Tris-Cl buffer. The results indicate an average of 3 measurements.

#### Antiviral Activity

Influence on microbial host and the antiviral activity of QCNCs was evaluated using biosafety level-1 (BSL-1) safe microbes *Escherichia coli* (*E. coli*), and nonenveloped bacteriophage PhiX174 (φX174) and MS2 as surrogates for more pathogenic mammalian viruses. MS2 is considered as suitable biosafe surrogate for evaluating antiviral activities as per US-EPA guidelines.^29^

##### Propagation of Phages and Host Organisms

Host of phage φX174, *E. coli*-C, was cultured on Luria–Bertani (LB) broth and LB-agar plates at 37 °C, whereas host of phage MS2, *E. coli*-M, was cultured in Medium-271 (ATCC) broth and agar plates containing streptomycin (2.0 mg/L). The phages were propagated as described elsewhere. Briefly, 100 μL of overnight cultured host organisms was added to 800 μL of LB broth supplemented with 20 mM of Ca^2+^ and Mg^2+^ ions (LB^+/+^), followed by adding 100 μL of phage stock culture (φX174 and MS2). The mixture was incubated for 10 min before adding to 3 mL of LB-agar (0.3% w/v) and cultured using a double layer agar (DLA) method. After overnight propagation, the plates were flooded with 5 mL of LB^+/+^ medium and placed in a shaker at 50 rpm at 28 °C for 4 h. The media from all the plates was pooled and centrifuged at 4500 rpm for 30 min to remove cell debris. The supernatant was filtered through a 0.22 μm syringe filter and stored at 4 °C. The purified phages were titrated by DLA-plaque forming units (PFUs) assay, to calculate the PFUs/mL present in the stock solution. The phages were not subjected to any further purification before using for virucidal/virus capture activity assay, to eliminate any chance for phage alteration or phage damage.

##### Antiviral Activity Analysis

Appropriate dilution of the functionalized CNCs during virucidal/virus capture activity assay was carried out, eliminating the effect of Q-CNCs on the host (*E. coli*) growth. For antiviral activity test, the viruses were dispersed at appropriate dilution in aqueous BSA solution (3 mg/mL) containing 20 mM of Ca^2+^ and Mg^2+^ ions (loading solution) (sterilized by 0.22 μm filtration). A 10-fold dilution of the viral stock solution, dispersed in loading solution/LB, represented a 10^–1^ dilution. Virucidal/virus capture activity was evaluated by suspension assay. Briefly, 200 μL of test material dispersed in sterile H_2_O containing 20 mM Ca^2+^ and Mg^2+^ ions and was taken in a 2 mL microcentrifuge tube; to this 10 μL of virus test dilution was added to eliminate any escape of virus from test material. The solution was incubated for 10 min before mixing thoroughly followed by further incubation for 24 h duration at 8 °C. After incubation period 790 μL of media was added to the tube to give 10^–2^ dilution.

The recovered viruses were serially diluted (10^–3^, 10^–4^, 10^–5^...) by diluting 100 μL of the recovered solution with 900 μL of respective medium, giving a 10-fold dilution to obtain individually separated countable plaques on a double layer agar (DLA) plate. 100 μL of overnight cultured host organisms was added to 900 μL of the above test dilution and incubated for 5 min. Subsequently, the inoculum was added to 3 mL of agar media (0.3% w/v) maintained at 50 °C in a 15 mL capped falcon tube, mixed thoroughly, and poured onto 90 mm agar plates for DLA assay. The plates were incubated at 37 °C for 24 h before testing for plaque formation. If required, the plates were further incubated for further 24 h for revised plaque counting. All tests were performed in duplicate each time and repeated at least four times (*n* = 8) to calculate average log reduction values. A compound that demonstrated logarithmic reduction of >4 log_10_ values in viral titers was considered virucidal/virus capture under the test conditions/concentrations.

## Results and Discussion

### Surface Cationization of CNCs

**Scheme 1:**
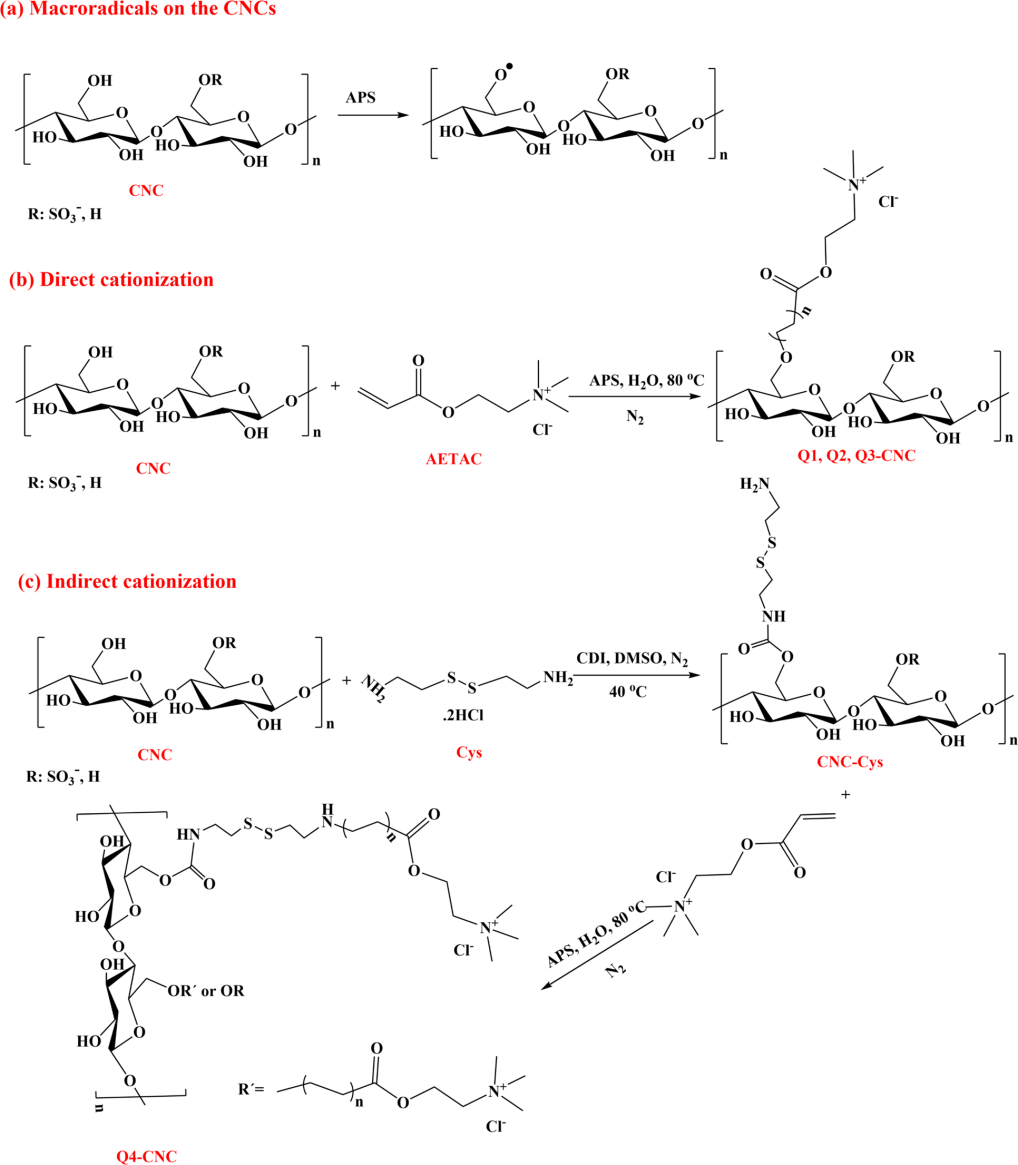
(a) Generation of Macroradicals on the CNC Backbone; (b) Direct Cationization, and (c) Indirect Cationization of CNCs

Polymer grafting onto CNC surfaces can be performed either by grafting to^30,31^ (grafting preformed polymers onto the CNCs surface) or grafting from^32,33^ (growing polymer chains from CNCs surface) approaches. In the grafting to method the grafting of long chain polymers may be limited by steric hindrance of long polymer chains, resulting in low density of polymer grafting. Because of that, as shown in Scheme 1, we used the grafting from method in which polymer chains can be grown directly from the CNCs surface through free radical polymerization. Scheme 1 illustrates the mechanism of grafting Q-PDMAEA onto CNCs surface by using water-soluble initiators for both direct and indirect grafting. In the presence of water-soluble persulfate initiators, (SO_4_^–·^) radicals get generated by thermal decomposition. These radicals produce active sites on the CNC surface hydroxyl groups, through which the vinyl monomers are covalently bonded to the backbone of cellulosic polymers (Scheme 1a).^34,35^ In the direct cationization (Scheme 1b), the hydroxyl groups of CNCs were modified using different ratios of AETAC monomer (Q1-, Q2-, Q3-) to the CNC by reaction of the CNCs macroradicals with AETAC monomer directly. Whereas via indirect approach (Scheme 1c), the hydroxyl groups of CNCs were first modified with Cys. This modification with Cys produce functional groups with flexible linker chains in addition to hydroxyl groups present on pristine CNC, enabling creation of higher number of available radical sites. AETAC could attach to both the free OH groups, and NH_2_ groups of Cys chains, resulting in high density of polymer grafting with AETAC (Q4-CNC). The main aim of using two methods for functionalization was to investigate the effect of degree of functionalization, charge density along with chain flexibility on physicochemical and antiviral properties of CNCs compared to direct graft polymerization.

**Figure 1:**
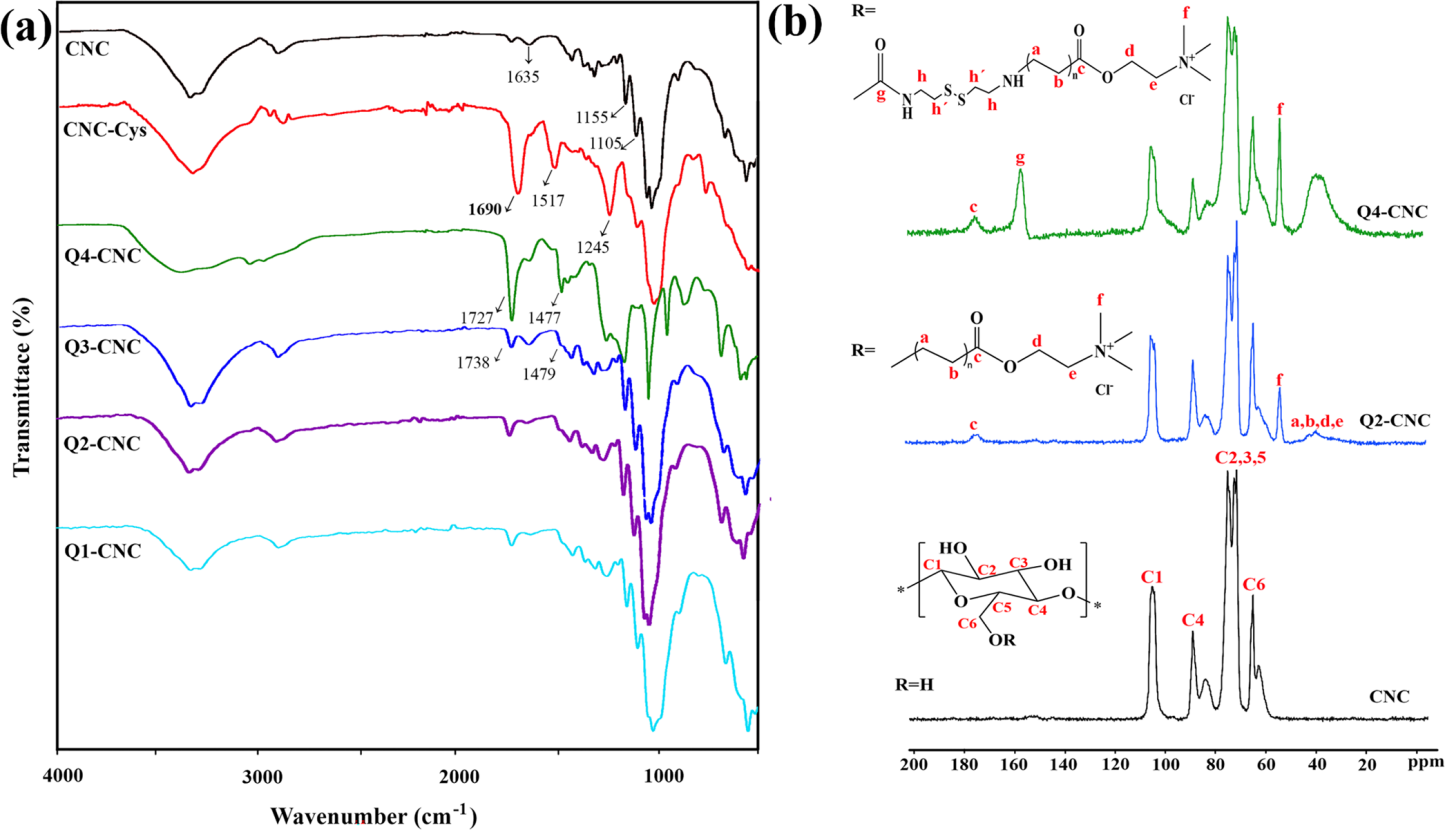
(a) FTIR spectra of CNC, CNC-Cys, and different types of Q-CNCs and (b) solid-state ^13^C NMR of CNC, Q2-CNC, and Q4-CNC.

The successful cationization of the CNCs was confirmed by FTIR, elemental analysis, and ^13^C NMR. Figure 1a showed the FTIR spectra of CNCs and QCNCs. In the FTIR spectrum of CNC the peaks at 3400 and 2890 cm^–1^ were attributed to the stretching vibrations of OH and CH groups, respectively. The peaks at 1635 cm^–1^, 1155 cm^–1^, and 1105 cm^–1^ were also assigned to the H–O–H bending vibration, C–C ring stretching band, and −C–O–C–vibrations, respectively.^36^ The spectra of all samples after modification and cationization showed the characteristic peaks of cellulose functional groups. Compared with CNC, the FTIR spectrum of CNC-Cys presented a new peak at 1690 cm^–1^ that was related to the stretching of the amide C=O groups, and the new peaks at 1517 and 1245 cm^–1^ were related to the N–H bending and C–N stretching, respectively. The results suggested that Cys has been attached to the CNC surface. In the FTIR spectrum of Q4-CNC, one characteristic peak was observed at 1727 cm^–1^, which was related to the carbonyl groups of urethane group. The presence of a quaternary ammonium group [−N^+^(CH_3_)_3_] attached on the CNC surface was confirmed by the peaks in the region of 1477 cm^–1^. According to the previous studies, the CH_2_ bending mode band at 1477 cm^–1^ was caused by methyl groups of the cationic substitution.^37,38^ As the stretching vibration band of S–S in Cys, CNC-Cys, and Q4-CNC was too weak to be measured by FTIR spectroscopy, Raman spectroscopy was used to detect this band. Figure S1 shows that the CNC did not have this band before modification at 502 cm^–1^. However, the presence of an S–S band of Cys after modification in CNC-Cys and Q4-CNC confirms surface CNCs modification.

The FTIR of Q1-, Q2-, and Q3-CNC predominantly showed a typical CNC spectrum with obvious two new peaks in 1738 and 1479 cm^–1^ that were related to the stretching of the C=O bond on AETAC chains and quaternary ammonium group [−N^+^(CH_3_)_3_], respectively, attached on the CNC surface.

Figure 1b displays the solid-state ^13^C NMR results of freeze-dried CNC, Q2-CNC, and Q4-CNC. All spectra showed characteristic peaks of the anhydroglucose units of cellulose,^16^ where the typical peaks corresponding to C1 (105.2 ppm), C4_cryst_ (89.2 ppm), C4_amorph_ (83.8 ppm), C6_cryst_ (65.4 ppm), and C6_amorph_ (62.7 ppm) were displayed.^39^ The C2, C3, and C5 showed relatively large resonance peaks between 70 and 80 ppm. After modification with AETAC in Q2-CNC, new peaks appeared in the regions of 40, 54.5, and 174.6 ppm, suggesting the graft polymerization of AETAC to the surface of CNCs. The peak at 54.5 ppm was attributed to the carbons of the trimethylammonium group (Cf) and the signal for carboxyl group was obvious at 174.6 ppm. The signals between 32 and 42 ppm were also due to the hydrocarbons in Ca, b, d, and e. In the Q4-CNC spectrum compared with the CNC, the presence of extra peaks at 176, 157.6, and 54.5 ppm and a broad peak at 40 ppm was associated with the carboxyl groups (Cc), urethane linkages (Cg), carbons of the trimethylammonium group (Cf), and hydrocarbon chains, respectively. Moreover, the degree of polymer grafting was also calculated based on the NMR results using peak integral of Cf (0.47 and 0.28 for Q4-CNC and Q2-CNC, respectively). The C1 signal was used as an internal standard and set to 1.

Elemental analysis was used to calculate the degree of substitution (DS) of CNC-Cys and degree of grafting polymer chains as well as confirming CNC surface modification and cationization (Table 1). The nitrogen content in CNC-Cys and Q-CNCs confirmed the successful grafting of Cys and AETAC on the surface of CNCs. Pure CNCs did not contain nitrogen and showed very small amount of sulfur, while the higher percentage of nitrogen and sulfur content in CNC-Cys and Q4-CNC confirmed the successful surface modification and quaterization of CNCs. The DS in CNC-Cys can be calculated by the following equations:^40^
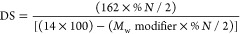
1where *N* represents the nitrogen content of modified CNC, 162 is the molecular weight of anhydroglucose unit, 14 is the molecular mass of nitrogen atom and *M*_w_ represents the net molecular weight of Cys (152.28 g/mol). Based on this equation, DS of CNC-Cys is 0.41.

**Table 1:** Elemental Analysis of CNC, CNC-Cys, and Different Types of Q-CNCs, Representing wt% of Different Elements

Sample	%C	%H	%N	%S	%O^a^	Polymer grafted
CNC	41.1	5.7	0	1.4	51.8	-
Q1-CNC	42.4	6.2	0.7	1.3	49.4	8.1
Q2-CNC	42.9	6.5	1.2	1.4	48.0	10
Q3-CNC	42.6	6.4	0.9	0.9	49.2	10
Q4-CNC	39.2	5.6	4.7	9.7	40.8	50
CNC-Cys	40.2	5.4	5.1	10.0	39.3	0.41^b^

a O% = 100% – (%C+%H+%N+%S).

b DS of Cys on CNC.

The weight percentage of QCNCs are calculated as described previously by Hemraz et al.,^4^ depicting that the weight ratio of polymer chains is 8.1%, 10%, 10%, and 50% for Q1-, Q2-, Q3-, and Q4-CNC, respectively. In Q1- and Q2-CNC, grafting of polymer chains appears to be improved by increasing the monomer ratio during the reaction. However, in Q3-CNC, the excess of monomer does not positively influence polymer chain weight ratio. This trend might be explained by the lower accessibility of the surface hydroxyl groups on CNCs for graft polymerization due to the hindered diffusion of the monomers. It is also possible that higher monomer concentrations can result in homopolymerization as a side reaction, therefore leading to lower grafting efficiency. However, the weight ratio of polymer chain in Q4-CNC is in accordance with the NMR results. When compared to other QCNCs, Q4-CNC showed the highest grafting ratio, which may be due to the unique method of grafting utilized (Scheme 1c) resulting in grafting from two sites.

### XRD and Surface Morphology

**Figure 2:**
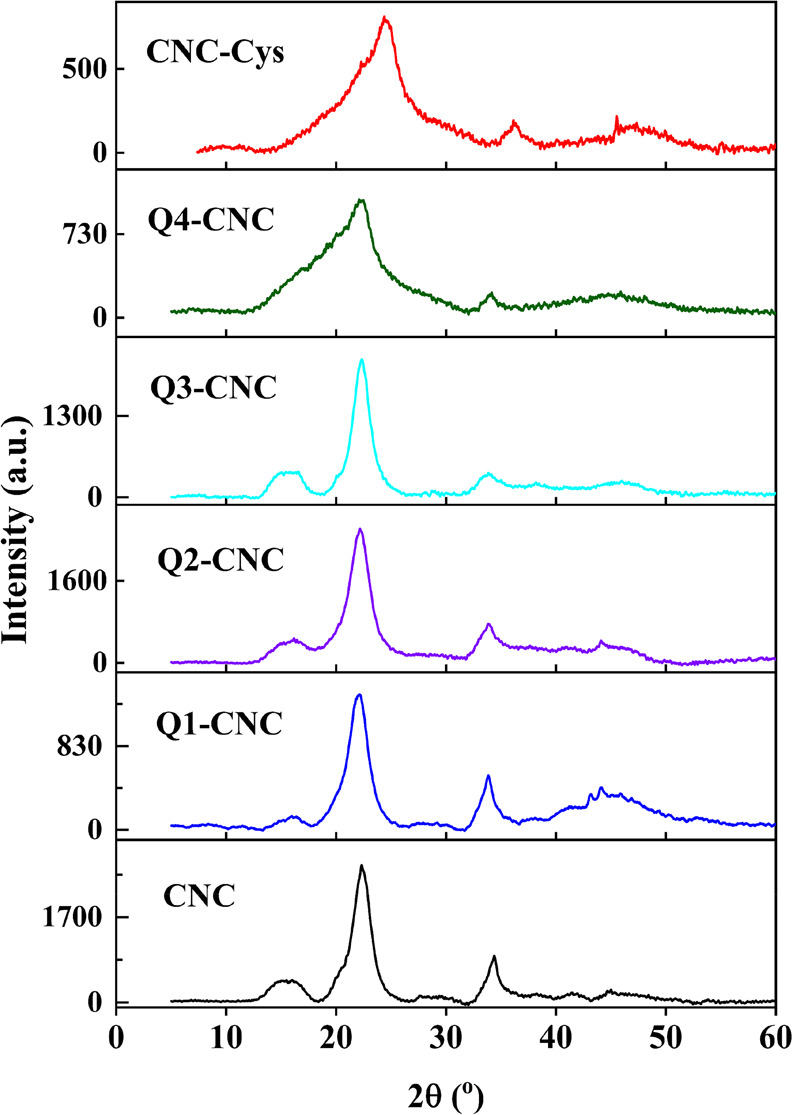
X-ray diffraction (XRD) patterns of CNCs, CNC-Cys, and different types of Q-CNCs.

Figure 2 shows the XRD patterns of CNCs, CNC-Cys, and different types of QCNCs. The CNCs exhibited characteristic peaks at 16.5° (11̅0), 22.5° (110), and 34.5° (200), corresponding to the cellulose I structure.^15,41^ The crystallinity index (CrI) of CNCs and QCNCs was calculated using Segal’s empirical equation (eq 2),^42^ where *I*_22.5°_ and *I*_18°_ represent diffraction intensity of crystalline and amorphous part of CNCs. The obtained results CrI for all samples are presented in Table S1.
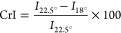
2

After the modification with Cys and cationization in Q4-CNC, the crystallinity index decreased from 98.5% for CNCs to 67.9%, 54.1% in CNC-Cys, and Q4-, respectively. Based on the XRD result, it is obvious that activation of hydroxyl groups on the CNCs surface with CDI and reaction with Cys can destroy the intermolecular and intramolecular hydrogen bonds leading to the change of CNCs’ crystalline region. In the case of Q4-CNC, forming the macromolecular layer on the surface of CNCs by radical polymerization might further change the molecular arrangement of the crystalline part or increase the amorphous content.^43^ Additionally, it demonstrates the successful grafting of the Cys linker and Q-PDMAEA onto the CNC. However, Q1-, Q2-, and Q3-CNC showed similar degrees of crystallinity with the crystallinity index of 95.3%, 89.1%, and 96.4%, which was slightly lower than pristine CNC (98.5%), representing a slight disruption in intra- and intermolecular hydrogen bonding.

The SEM micrographs of CNC, CNC-Cys, and Q-CNCs are shown in Figure S2. Pristine CNCs showed a very smooth appearance in SEM with some individual needle-like crystallites. However, after direct cationization (Q1-CNC, Q2-CNC, and Q3-CNC), the morphology of the QCNCs was changed to significantly rougher with an increase in AETAC ratio. The SEM image of CNC-Cys and Q4-CNC compared to CNC showed rougher surface and more aggregation.

**Figure 3:**
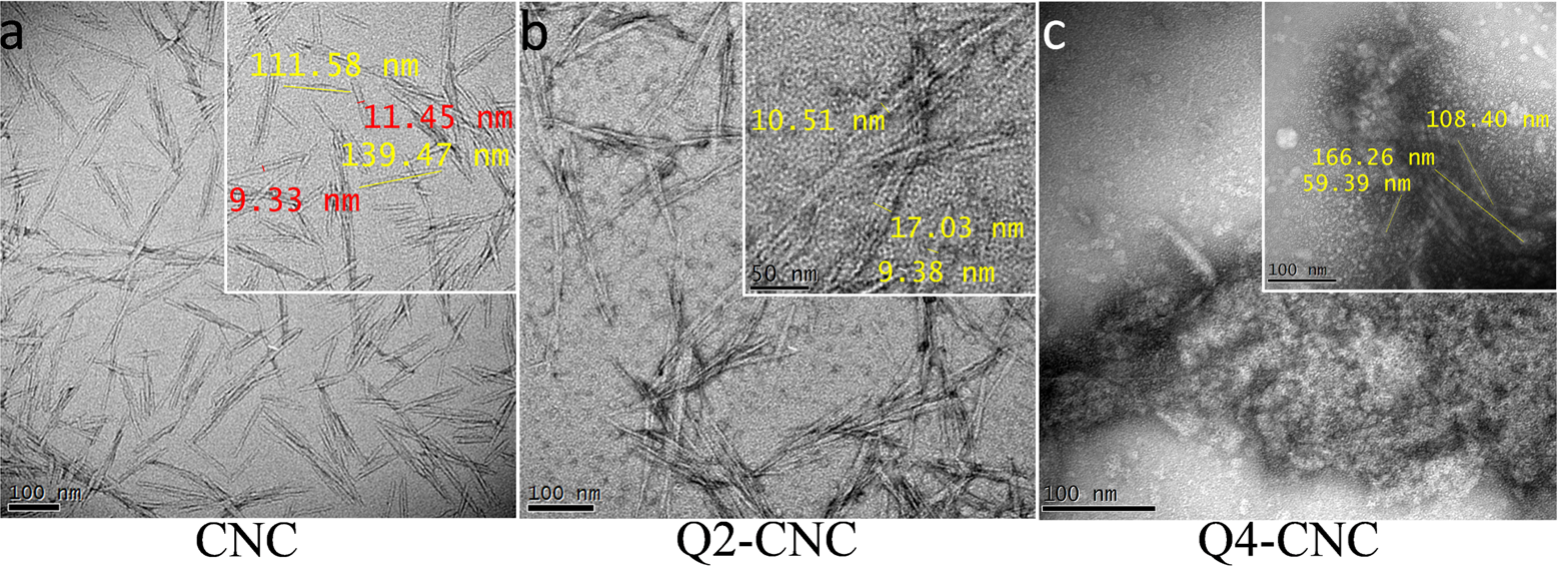
TEM images representing (a) CNC, (b) Q2-CNC, and (c) Q4-CNC showing size, shape, and nature of the materials. (Scale bar = 100 nm.)

The TEM images of CNCs, Q2-CNC, and Q4-CNC are shown in Figure 3. As shown in Figure 3a, the CNCs have rod-like shape with 110 ± 30 nm length and width of 10 ± 2 nm. After direct graft polymerization, the size or shape of CNCs was not affected in Q2-CNC. However, in indirect graft polymerization the width and rod-shape of CNCs had changed in Q4-CNC. In Q4- the CNC crystals were found surrounded by polymer matrix showing a higher amount of polymer chains present on crystal surfaces. Similar results were demonstrated by XRD results; Q4-CNC’s crystal form appears to be more amorphous, which indicates that chemical modifications have changed the crystal form of CNCs (Figure 2).

The morphology and structure of CNCs, Q2-CNC, and Q4-CNC was further observed by AFM. As shown in Figure S3, a large number of aggregates can be observed in the CNCs image. This may be caused by the fast evaporation of water molecules inducing agglomeration of CNCs and leading to more CNCs agglomeration. AFM images of Q2-CNC and Q4-CNC (Figure S3) support the observations made above from the TEM images.

### Thermal Stability

**Figure 4:**
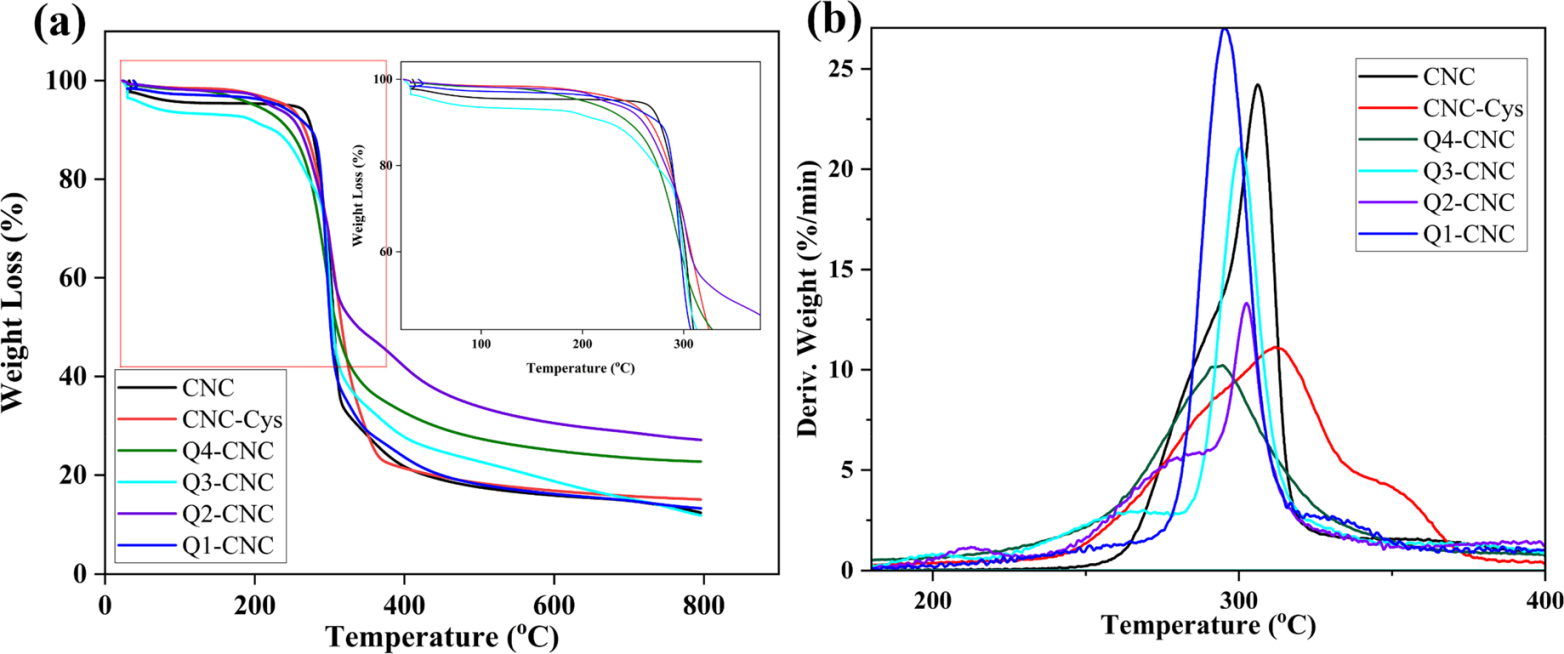
(a) Thermogravimetric and (b) differential gravimetric curves of CNCs, CNC-Cys, and different types of Q-CNCs.

The weight loss curves of the samples are shown in Figure 4, and results obtained from the thermograms, including the decomposition temperature at 50% weight loss (T50) and samples’ char at 800 °C, are reported in Table 2. Pure CNC shows two distinct pyrolyses like the typical degradation patterns for CNCs containing sulfate groups.^44^

**Table 2:** Thermal Analytical Data of CNC, CNC-Cys, and Q-CNCs

Samples	*T*_50_	Char (%)
CNC	306	12.4
Q1-CNC	301	13.2
Q2-CNC	338	27.1
Q3-CNC	304	12.0
Q4-CNC	309	22.7
CNC-Cys	316	15.1

There was a three-stage weight loss for CNC-Cys, and all the QCNCs had an initial weight loss of approximately 4–5% upon heating to 100 °C, which was attributed to the vaporization and loss of moisture. Two other weight losses result from pyrolysis of hydrocarbon chains.^45^ Significant decomposition was observed at the second stage at the temperature range of 300–310 °C for all samples, CNCs, CNC-Cys, and Q-CNCs. The initial decomposition temperature and decomposition temperature at 50% weight loss (T50) are indicative of thermal stability. Q2-CNC possessed a higher thermal stability than CNC and other QCNCs.

By comparing the weight residual content of samples at 800 °C, it was obvious that all QCNCs showed higher weight residual content than CNCs. Therefore, it can be concluded that the surface of CNCs is wrapped by covalently bonded thin polymer layer, resulting in delay of pyrolysis and an increase of char. A shift in peak derivative weight toward lower temperature (Figure 4b) for Q-CNCs compared to pristine CNC also demonstrates decrease thermal stability due to loss of inter- and intramolecular hydrogen bonding because of functionalization.

### Dispersion of QCNCs

**Figure 5:**
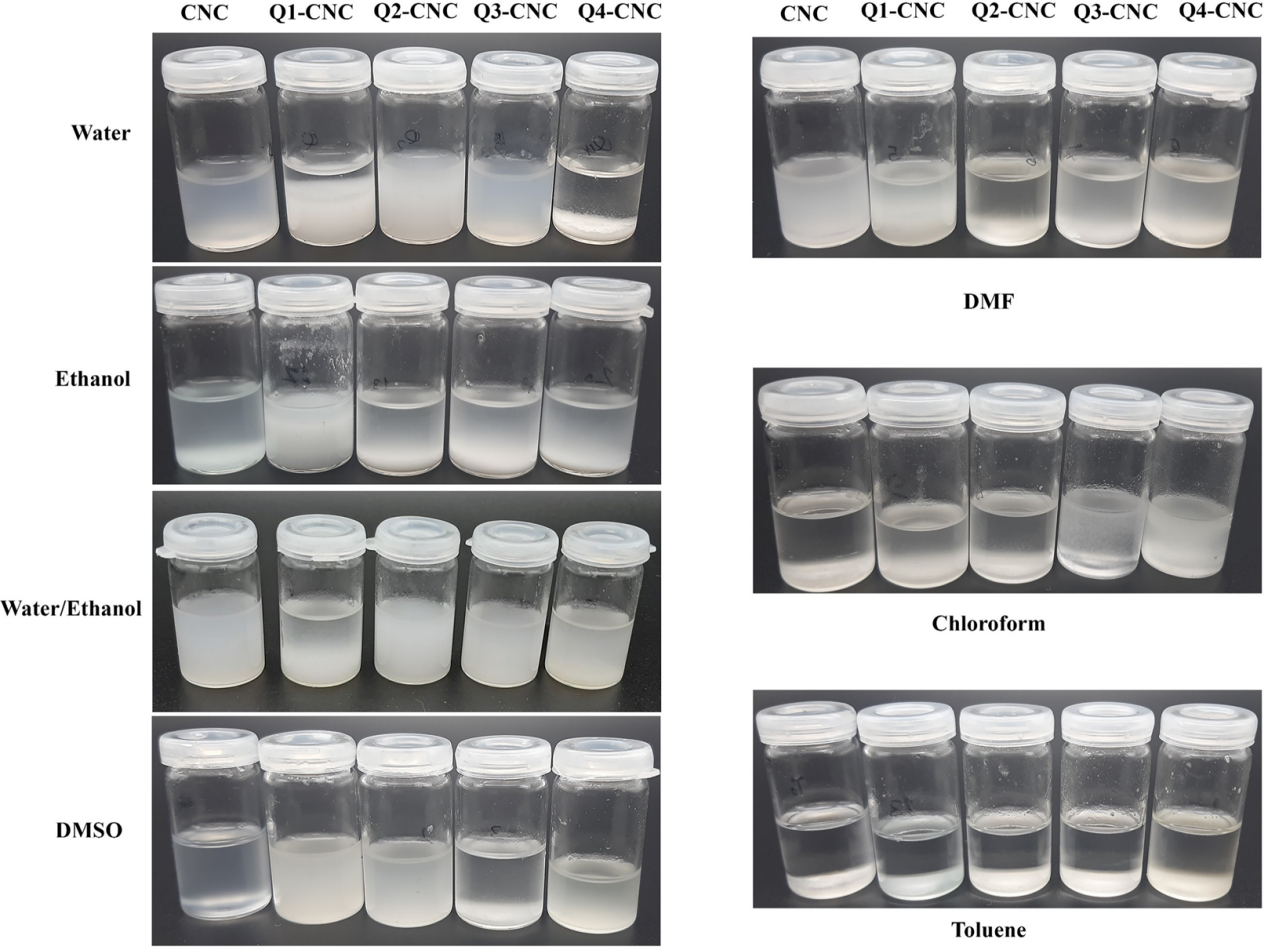
Dispersibility of CNC and QCNCs in various media 30 min after redispersion.

The redispersion of freeze-dried CNCs and QCNCs in water and other organic solvents (DMF, ethanol, dichloromethane, and toluene) was also investigated (Figure 5) in this study. The CNC and directly functionalized QCNCs showed a stable suspension in water, whereas Q4-CNC was difficult to disperse in water even after sonication. However, the Q4-CNC dispersed better in ethanol and even very well in the mixture of water/ethanol. This might be due to the high grafting ratio of polymer chains in Q4 leading to hindrance in interaction with water molecules. The hydrocarbon portion of ethanol may be able to form van der Waals forces with the hydrophobic portion of polymers in an ethanol/water mixture, leading to improved dispersion of modified CNC in the water–ethanol mixture. As shown in the Figure 5, all the samples showed good dispersion in DMSO. In the nonpolar solvent like chloroform and toluene neither CNC nor any of the QCNCs dispersed well, precipitating within 5 min postdispersion.

### Surface Charge and Particle Sizes

The hydrodynamic size (*D*_h_) distribution and ζ-potentials of CNCs, CNC-Cys, and Q-CNCs were determined by a DLS particle size analyzer. Based on DLS results (Table 2S), the *D*_h_ of CNC was about 60 nm while QCNCs with direct cationization showed larger *D*_h_. In fact, the *D*_h_ of QCNCs increased by increasing the weight ratio of monomer (Q1 < Q2 < Q3) demonstrating a higher number of quaternary groups present on the surface. CNC-Cys also showed larger size compared with CNCs with *D*_h_ of 164 nm. This increase in *D*_h_ showed that the CNC surface was successfully modified with Cys. However, Q4-CNC depicted the largest *D*_h_. The reason for this is the higher density of grafts in Q4-CNC compared to other QCNCs along with longer and flexible polymer chains.

ζ-Potentials of pristine CNCs and QCNCs was also characterized by DLS in Milli-Q (pH = 5.5) (Table 2S). The Pristine CNCs showed negative ζ-potential of −22.2 mV, due to presence of anionic sulfate ester groups. Q-CNCs, on the other hand, showed positive ζ-potentials. These data confirmed the presence of positively charged groups on the CNCs surface. In addition, the ζ-potential values of CNC, CNC-Cys, Q4-CNC, and Q2-CNC at different pH values are shown in Table 3. In case of CNC, at all pH ranges the ζ-potential was negative because of existing sulfate ester groups (OSO_3_^–^) on the surface of CNC. Negative charges are created during sulfuric acid hydrolysis at acidic pH values. While CNC-Cys possessed positive ζ-potentials at low pH due to the protonation of free amine groups, on increasing the pH the ζ-potential values became negative owing to the deprotonation of amine groups present on Cys. On the other hand, after cationization with AETAC and inserting permanent positive charges arising from [−N^+^(CH_3_)_3_] groups, relatively positive ζ-potential were observed for Q1-, Q2-, and Q3-CNC over a large pH range (pH 2–10). Q4-CNC showed the less negative ζ-potential compared to CNCs and CNC-Cys after graft polymerization with cationic monomer as CNC particles were largely embedded in amorphous polymeric matrix.

**Table 3:** Table Representing Surface Charge (ζ) on CNC and QCNCs under Different pH Conditions

Material	HCl (pH 2.0)	Phosphate buffer (pH 6)	Phosphate buffer (pH 7.4)	NaOH pH 8	NaOH pH 10
CNC	–29.2 ± 0.5	–17.9 ± 0.6	–13.8 ± 1.68	–40.4 ± 0.9	–37.3 ± 1.4
Q1-CNC	17.8 ± 0.9	4.81 ± 0.2	0.36 ± 0.3	–21.2 ± 1.7	–25.4 ± 0.6
Q2-CNC	30 ± 1.4	16.1 ± 0.1	7.52 ± 0.1	3.16 ± 0.9	–13 ± 0.3
Q3-CNC	26.6 ± 2.1	14.8 ± 0.9	7.4 ± 0.6	5.0 ± 0.1	–8.3 ± 1.6
Q4-CNC	27.7 ± 2.0	–11.0 ± 0.5	–10.2 ± 0.9	–27.4 ± 0.7	–32.2 ± 0.2
CNC-Cys	0.7 ± 0.3	–13.5 ± 0.8	–20.6 ± 0.3	–25 ± 1.9	–27.6 ± 0.3

### Antiviral Activity

**Figure 6:**
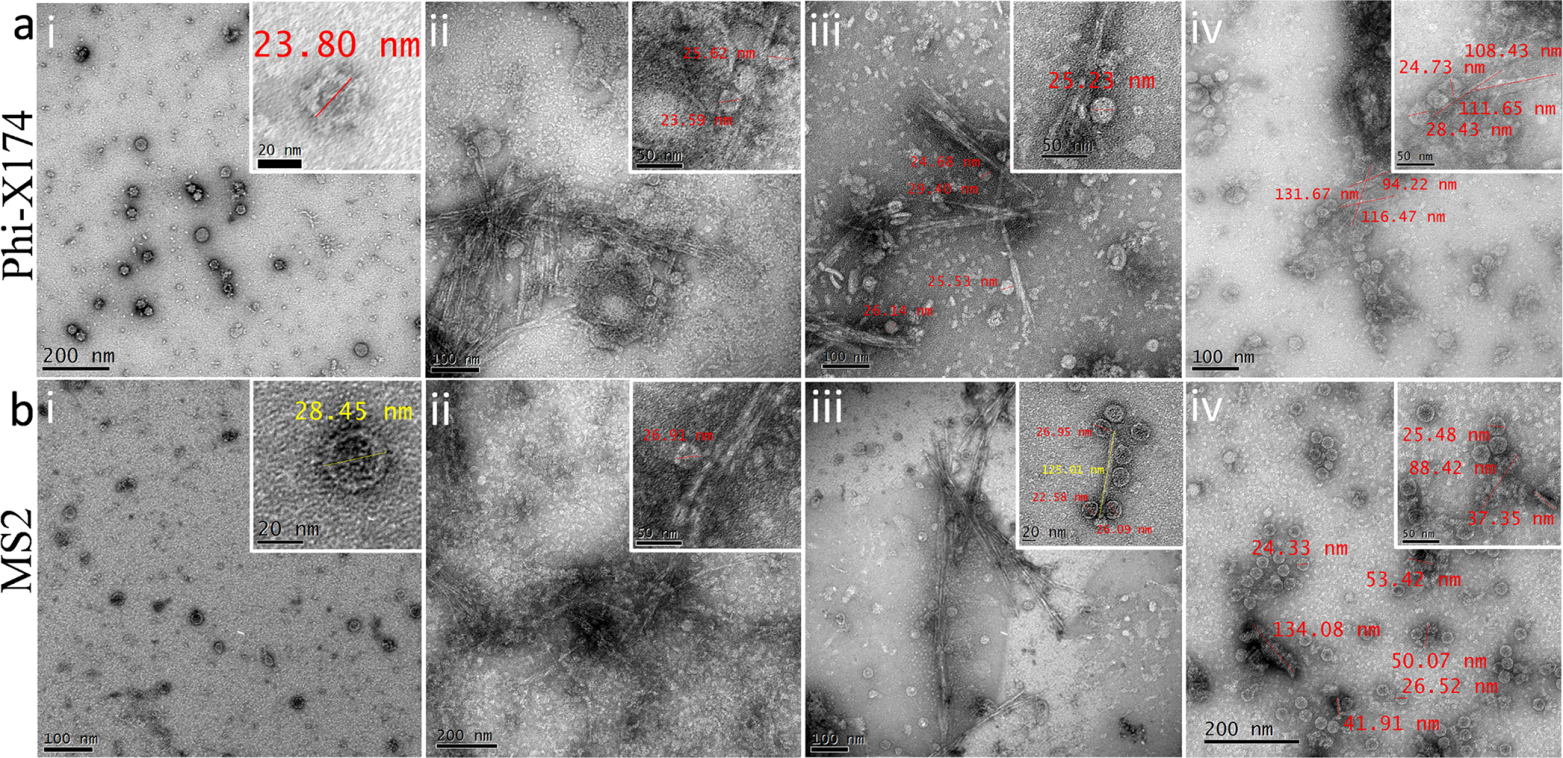
TEM images showing size and structure of (a)(i) φX174 and (b)(i) MS2 and their interaction with (ii) Q1-CNC, (iii) Q2-CNC, and (iv) Q4-CNC, respectively. Interaction between virus and cationized CNCs demonstrated.

The results demonstrated that neither CNC nor CNC-Cys were able to significantly reduce viral loads under test conditions. Comparatively Q-CNCs demonstrated significant virucidal/virus capture activity, although the activity varied from virus to virus. The virucidal/virus capture activity of the materials differed for both φX174 and MS2. φX174 was more susceptible to the functionalized CNCs; Q-CNCs were more effective in eliminating φX174 to significantly higher levels, whereas they were not as effective in neutralization of MS2, which appeared to be more resistant toward the treatment and difficult to eliminate (Figure 6). A lower net negative charge on φX174 compared to MS2 (Table 4) along with lower aggregation of QCNCs can be the reason for higher binding. It was observed that virus particles adhered to the surface of functionalized CNC crystals. A higher density of viral particles can be observed near to the CNC crystals in comparison to the background demonstrating adhesion. Further, it was observed that the functionalized materials also contained some amorphous polymer chains bound to CNC crystals in functionalized QCNCs, which were also interacting with viral particles (Figures 6 and S4). The amount of this amorphous component was higher in Q4-CNC compared to Q1-, Q2-, and Q3-CNC. This binding mechanism can be responsible for the virus capture activity of different materials against φX174 and MS2. The overall virus capture activity can be the result of combined binding with the functionalized CNC particles and the amorphous polymeric matrix present.

**Table 4:** ζ-Potential Values of Viruses Studied in Different Environments

Virus	Average ZP (mV) pH 6.0 (PB)	Average ZP (mV) pH 7.0 (Tris-Cl)
φX174	–14.63	–8.80
MS2	–12.03	–7.75

**Figure 7:**
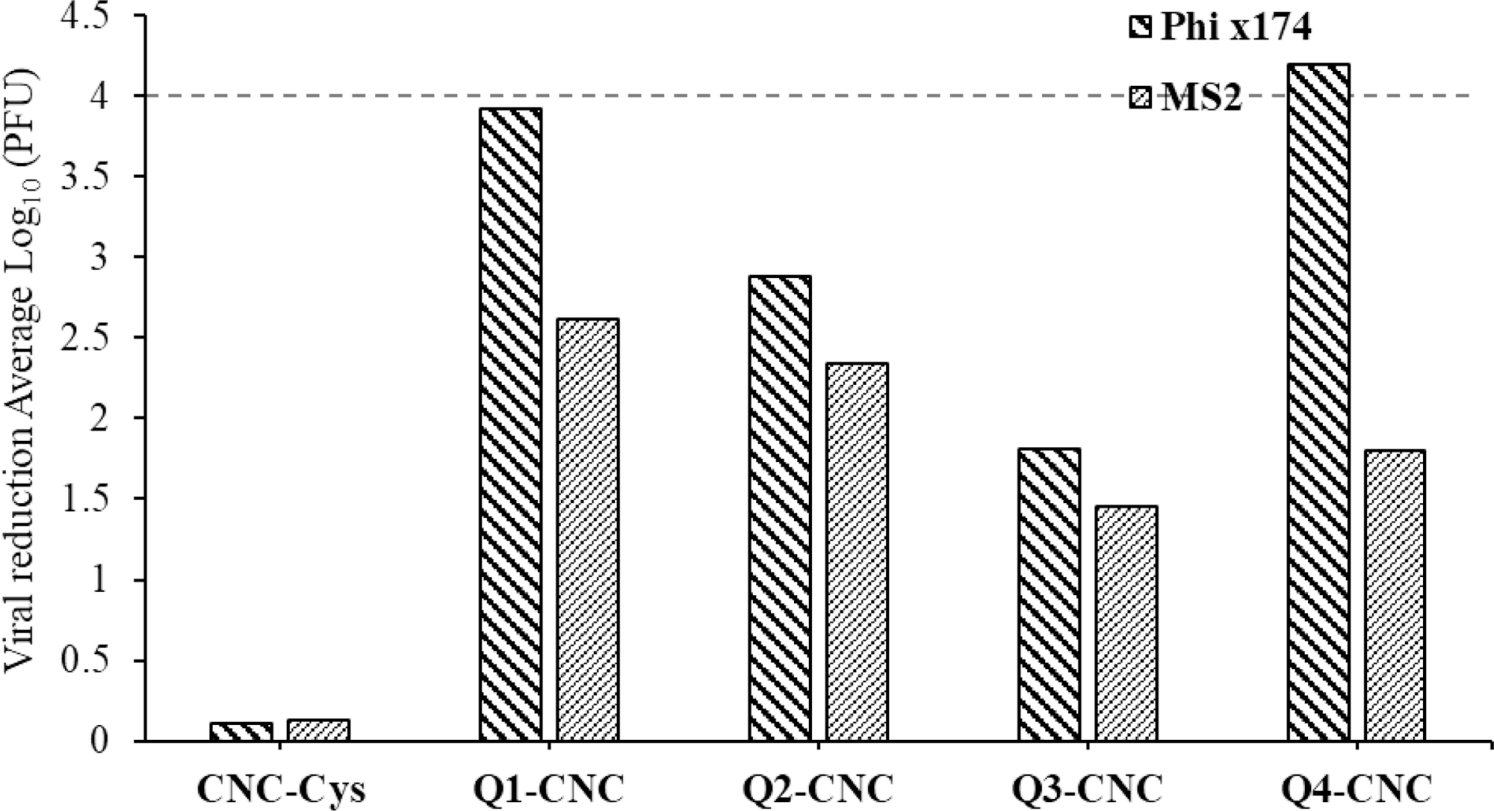
Graph showing virucidal/virus capture activity of different materials against φX174 and MS2 viruses. Viral reduction >4 log_10_ values (dashed line) is considered significant viral reduction activity. (*n* = 8.)

Here Q4-CNC which includes a chain extender linker (Cys) between CNC and QCNC demonstrated much higher activity compared with other CNCs where direct surface functionalization was performed, and functional groups were present much closer to the functionalized surface. Q4-CNC had highest viral reduction against φX174 (log_10_ 4.19), whereas only a 1.80 log_10_ reduction of MS2 was observed. The next highest activity was demonstrated by Q1-CNC against both φX174 (log_10_ 3.92) and MS2 (log_10_ 2.61) virus (Figure 7). However, a decrease in overall virucidal/virus capture activity was demonstrated with an increase in the degree of cationization (functional group density) on the CNC surface, where Q2-CNC demonstrated a reduction by 2.87 and 2.34 log values and Q3-CNC had reduction by 1.81 and 1.45 log values for φX174 and MS2, respectively (Figure 7). A viral reduction of >4 log_10_ values was considered a significant viral reduction property. This decrease in the activity can be attributed to the emergence of reduced degrees of freedom and steric hindrance emerging due to direct functionalization, as the functional groups are present much closer to the surface. This can also be observed as a larger hydrodynamic radius of Q4-CNC in DLS, due to longer chains, enhancing the hydrodynamic radius compared to smaller hydrodynamic radius of the Q1-, Q2-, and Q3-CNCs and CNC-Cys. Further, with an increase in the monomer ratio Q1 < Q2 < Q3, a decrease in the overall virucidal/virus capture activity was observed. This can again be attributed to higher aggregation of the materials due to the higher amorphous component of the polymeric functional groups (higher cationization) on the surface leading to hindered interaction with both water molecules and the virus.

## Conclusions

Cationic Q-PDMAEA polymer grafted CNCs were prepared in two ways by direct and indirect graft polymerization. Several measurements, such as FTIR, NMR, elemental analysis, and DLS, confirmed the successful preparation of the QCNCs with polymer chains attached either directly to the CNC surface or via a linker for enhanced chain flexibility. Cationization of the CNC provides positive charge to the CNC surface, enabling charge mediated interactions with oppositely charged virus particles. Such functionalization can lead to higher virus capture, disabling them from interacting with the host organism or neutralizing them. Additionally, it was observed that virus elimination activity is not directly related with surface charge density. Higher surface functionalization led to hindrance in the interactions decreasing the antiviral activity, whereas an increase in the chain flexibility due to presence of a linker increases the antiviral activity. The virus elimination efficacy also depends on the virus surface properties and surface charge present on the virus particle. Therefore, the inhibition activity also varies from virus-to-virus. The results demonstrate that such cationically charged polymeric matrices or surfaces efficiently capture viruses. In summary, direct and indirect cationization of CNCs opens up new possibilities for utilizing CNCs as biobased antiviral agents in textile, packaging, wastewater treatment, and medical applications.

## Supporting Information Available

Raman spectra, crystallinity index of CNC and modified CNC’s, SEM image of CNC matrices, AFM images of the CNC and modified CNC’s, ζ-potential and hydrodynamic radius determined with DLS, TEM images showing interaction of φX174 with modified CNC (PDF)

## Abbreviations

AETAC: [2-(acryloyloxy)ethyl] trimethylammonium chloride
APS: ammonium persulfate
CNCs: cellulose nanocrystals
CDI: 1,1′-Carbonyldiimidazole
DLS: dynamic light scattering
DMSO: dimethyl sulfoxide anhydrous
FT-IR: Fourier transform infrared spectroscopy
QCNCs: cationization CNCs with Q-PDMAE
Q-PDMAEA: poly(2-dimethylamino)ethyl acrylate) methyl chloride quaternary salt
XRD: X-ray diffraction spectroscopy
SEM: scanning electron microscopy
TEM: transmission electron microscopy
TGA: thermogravimetric analysis
