# Supplementary material for: Direct and Indirect Cationization of Cellulose Nanocrystals: Structure–Properties Relationship and Virus Capture Activity
Source: Biomacromolecules. 2022 Dec 5;24(10):4397–407. doi: 10.1021/acs.biomac.2c01045 (PMC10565721; doi:10.1021/acs.biomac.2c01045)
Supplement: Supplementary file 1 — bm2c01045_si_001.pdf [file bm2c01045_si_001.pdf]

SUPPORTING INFORMATION

Direct and indirect cationization of cellulose nanocrystals: structure properties relationship and virus capture activity

*Maryam Madani, Sedigheh Borandeh, Arun KumR Teotia, Jukka V. Seppälä**

Polymer Technology, School of Chemical Engineering, Aalto University, Kemistintie 1, Espoo, 02150 Finland

Supplementary Figures:

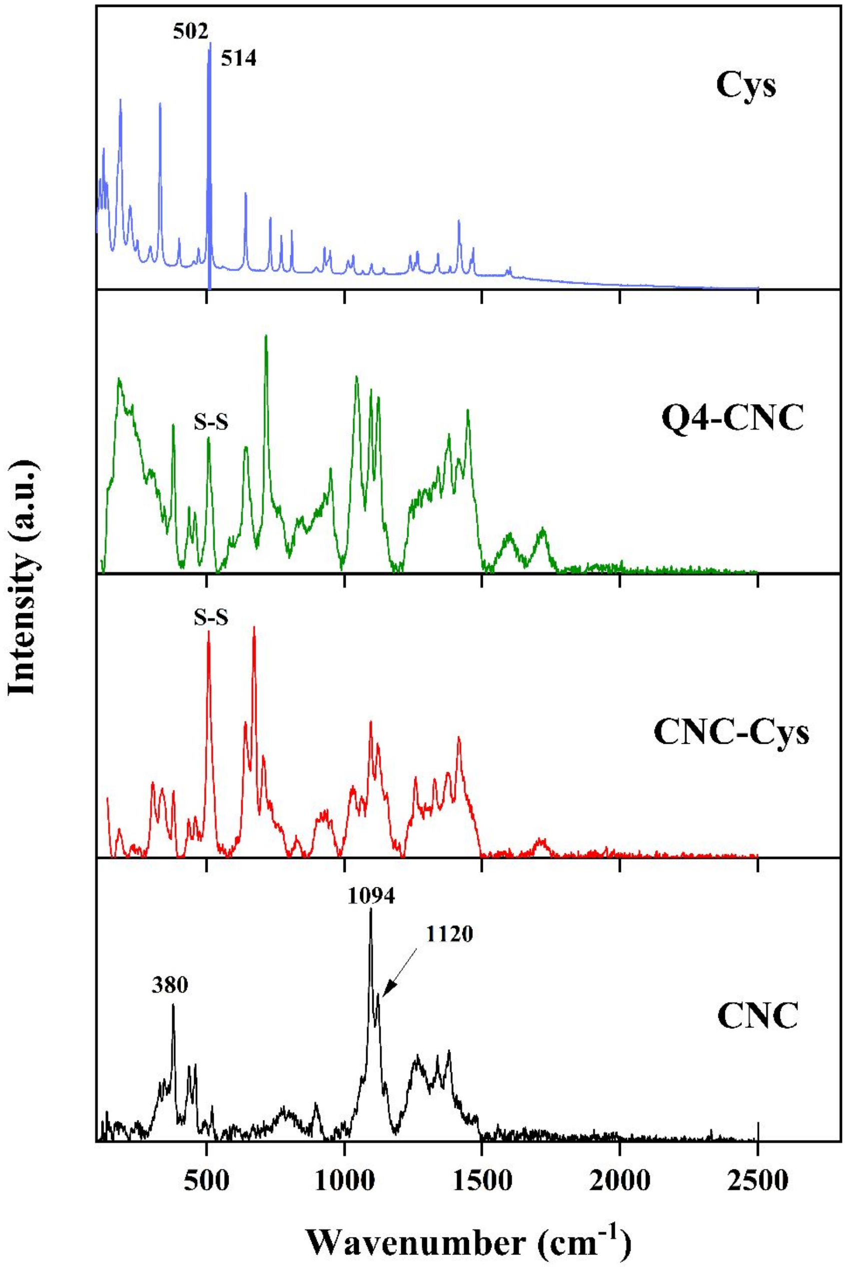

Figure S1: Raman spectra of CNCs, CNC-Cys, Q4-CNC and Cys.

Table S1. The crystallinity index (CrI) of CNCs and different types of QCNCs.

Samples	$I_{22.5^{\circ}}$	$I_{18^{\circ}}$	CrI
CNC	2763.1	259.6	90.6
Q1-CNC	1301.7	118.9	90.8
Q2-CNC	2354.3	281.1	88.0
Q3-CNC	2223.1	268.3	87.9
Q4-CNC	1007.1	462.4	54.1
CNC-Cys	466.3	149.3	67.9

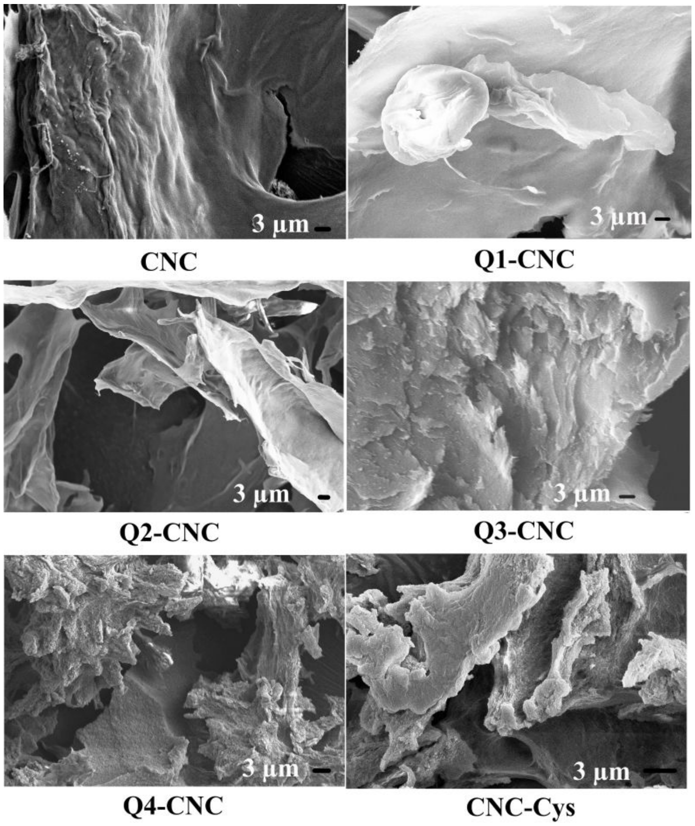

Figure S2. SEM images of CNC, Q1-, Q2-, Q3-CNC, Q4-CNC, and CNC-Cys. (Scale bar = 3μm)

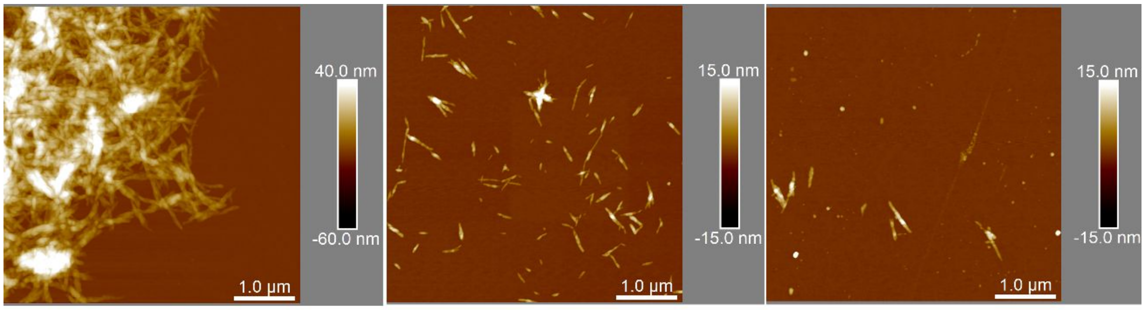

CNC **Q2-CNC** **Q4-CNC**

Figure S3: AFM images of CNC, Q2-CNC and Q4-CNC

Table S2: Hydrodynamic diameter and ζ-potentials values of CNC, CNC-Cys and Q-CNCs by DLS.

Sample	D_h (nm)	ζ-potentials (mV)
CNC	60	-22.2±0.8
Q1-CNC	79	16.5±1.0
Q2-CNC	176	18.6±1.8
Q3-CNC	182	18.7±0.9

Q4-CNC	249	11.5±1.3
CNC-Cys	164	-15.62

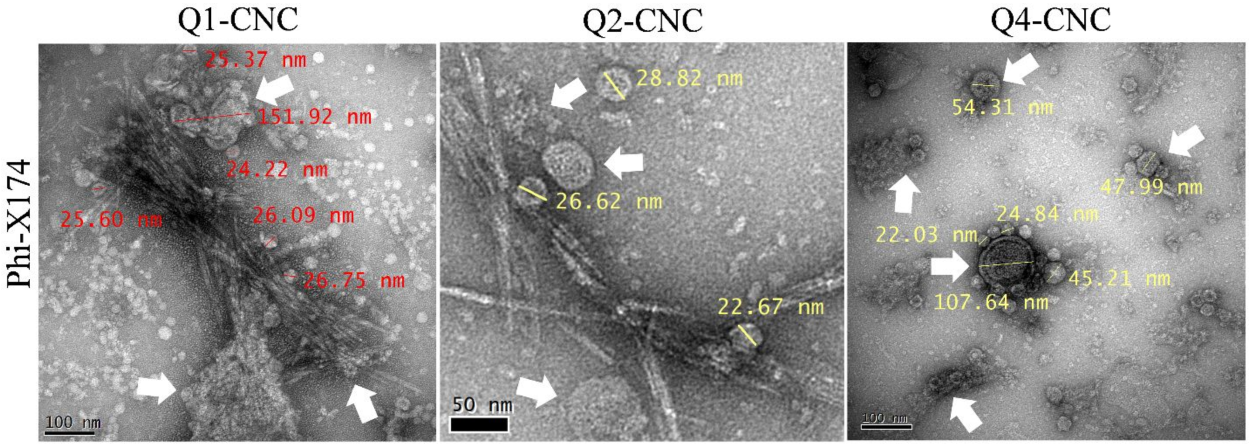

Figure S4: TEM images of Q1-, Q2- and Q4-CNC. Showing interaction of Phi-X174 with functionalized CNC and the amorphous polymeric matrix present (white arrows).
